# Molecular identification of *Bursaphelenchus cocophilus* associated to oil palm (*Elaeis guineensis*) crops in Tibu (North Santander, Colombia)

**DOI:** 10.2130/jofnem-2020-117

**Published:** 2020-11-30

**Authors:** Greicy Andrea Sarria, Donald Riascos-Ortiz, Hector Camilo Medina, Yuri Mestizo, Gerardo Lizarazo, Francia Varón De Agudelo

**Affiliations:** 1 Pests and Diseases Program, Cenipalma, Experimental Field Palmar de La Vizcaína, Km 132 Vía Puerto Araujo-La Lizama, Barrancabermeja, Santander, 111611, Colombia; 2 Facultad de Agronomía de la Universidad del Pacífico, Buenaventura, Valle del Cauca, Campus Universitario, Km 13 vía al Aeropuerto, Barrio el Triunfo, Colombia; 3 Extension Unit, Cenipalma, Tibu Norte de Santander, 111611, Colombia

**Keywords:** *Bursaphelenchus cocophilus*, Diagnostic, Molecular biology, Phylogenetic analysis

## Abstract

The red ring nematode (*Bursaphelenchus cocophilus* (Cobb) Baujard 1989) has been registered in oil palm crops in the North, Central and Eastern zones of Colombia. In Tibu (North Santander), there are doubts regarding the diagnostic and identity of the disease. Oil palm crops in Tibu with the external and internal symptoms were inspected, and tissue samples were taken from different parts of the palm. The refrigerated samples were carried to the laboratory of Oleoflores in Tibu for processing. The light microscopy was used for the quantification and morphometric identification of the nematodes. Specimens of the nematode were used for DNA extraction, to amplify the segment D2-D3 of the large subunit of ribosomal RNA (28S) and perform BLAST and a phylogeny study. The most frequently symptoms were chlorosis of the young leaves, thin leaflets, collapsed, and dry lower leaves, beginning of roughening, accumulation of arrows and short leaves. *Bursaphelenchus*, was recovered in most of the tissues from the samples analyzed: stem, petiole bases, inflorescences, peduncle of bunches, and base of arrows in variable populations. The morphometric data and sequences obtained for the segment D2-D3 confirms to *B. cocophilus* as the causal agent of red ring disease on oil palms in the study area. For our knowledge, this study reports the first partial sequences of segment D2-D3 of *B. cocophilus* in oil palm in Colombia.

The oil palm industry is one of the most important in the Colombian agricultural sector. Currently, Colombia is the fourth producer of palm oil and the first in Latin America, with more than 535,000 hectares planted in 112 towns of 20 states, placing it as one of the main agricultural lines of the country (Fedepalma, 2018; Sistema de información estadística del sector palmero-SISPA, 2019). One of the most important aspects of Colombian palm production is related to phytopathological problems. These diseases are considered as the main threat and have been responsible for crop losses, including the so-called lethal ones such as sudden wilt (*Phytomonas* sp.), lethal wilt (unknown etiology), and the red ring disease associated with the migratory endoparasite nematode *Bursaphelenchus cocophilus* (Cobb, 1919; Goodey, 1960; Baujard, 1989) (=*Rhadinaphelenchus cocophilus* [Cobb]) (Martínez, 2010).

The red ring disease has been reported in Central America (Guatemala, Nicaragua, Belize, Costa Rica, El Salvador, Honduras, Mexico, Panama), Latin America (Brazil, Ecuador, Guyana, French Guyana, Peru, Venezuela, and Colombia), and in the southern Caribbean (Granada, San Vicente, Tobago, Suriname, Trinidad, Dominican Republic) (Kraaijenga and den Ouden, 1966; Kastelein, 1987; Giblin-Davis, 2001; Sánchez and Cerda, 2002; Brammer and Crow, 2002a).

In Colombia, there is a history of the red ring since 1960s. In 1967, more than 10,000 ha of coconut palm disappeared on the Pacific coast. In 1986, red ring disease was detected in oil palm in Palmeras de la Costa plantation (El Copey, state of Cesar) and currently, in the Eastern Plains and the state of North Santander, causing in addition to the red ring, the short leaf syndrome (Varón de Agudelo and Granada, 1986; Chinchilla, 1992; Calvache et al., 1995; Aldana et al., 2015).

The red ring disease has caused losses that reached 8 million dollars between 1990 and 2002 in the North and Eastern production areas (Aldana et al., 2015). It has been registered mainly in crops from North, Central, and Eastern zone of Colombia, where management strategies are mainly preventive through frequent monitoring and trapping of the *Rynchophorus palmarum* nematode vector (Coleoptera: Curculionidae) (Blair and Darling, 1968; Griffith, 1969; Oehlschlager et al., 2002). Other management practices include early detection and timely eradication of plants, followed by proper waste disposal (Van Hoof and Seinhorst, 1962; Griffith, 1987; Chinchilla, 1992; Chinchilla and Escobar, 2007; Saenz, 2016).

The red ring symptoms in oil palm are varied in Colombia, in the case of the Eastern Plains the main symptom is the shortening of leaves. In the North Zone, there is shortening and copper coloration of the leaflets. In some cases, there is accumulation of spear leaf and when they open are very short and chlorotic (Chinchilla, 1992; Calvache et al., 1995). However, external symptoms are not considered a diagnostic method of the disease because there are other pathogens that induce similar symptoms in the foliage (Dean, 1979). The most frequent symptoms are young leaf chlorosis, drying of lower leaves, breaking of petioles, and leaf folding. Internally in the petioles and spear leaf, brown or reddish brown spots may be observed, and in the stem continuous or discontinuous, brown or reddish brown rings (Brathwaite and Siddiqi, 1975; Chinchilla, 1992; Calvache et al., 1995; Brammer and Crow, 2002b). The final state of the disease is the death of plants (Thorne, 1961; Giblin-Davis, 1990; Sáenz, 2005; Giblin-Davis et al., 2010).

*Bursaphelenchus cocophilus* can be found infecting the parenchymal tissues of the stem, petioles and sometimes in roots, the intercellular spaces, and in an advanced state of the disease, they act as intracellular parasites (Griffith, 1987; Griffith and Koshy, 1990), causing the interruption of the transport of nutrients, water, and sap, which induces drying of the leaves. In palms with short leaves, the nematodes are found mainly in the leaf buds, which makes the palm generate shorter, deformed, yellow, and dry leaves (Brammer and Crow, 2001; Oehlschlager et al., 2002). It have been reported that in raining season the nematode populations increase and they move toward arrows, central leaves, tissue in the leaf buds, and the spear leaf (Volcy, 1998) and *B. cocophilus* achieves the central arrow may access to any petiole (Menjivar et al., 1988; Cuthbert, 1993; Ferris, 1999).

Morphologically, males of *B. cocophilus* are characterized by having sclerotized cephalic region, paired spicules, absent gubernaculum, a conoid to sharply pointed tail, and strongly ventrally curved, which contains a terminal bursa, presents two pairs of ventrosubmediales papillae very close at the base of the bursa and a pair preanal, anterior to the cloacal aperture (Opperman, 1995). The females are characterized by having a thin cuticle, marked with transverse striations, lateral fields with four incisures, the labial region is high and smooth, covered vulva by a protuding anterior vulvar lip, presence of a long post-uterine sac, a narrow and elongated tail with a clindroid and rounded terminus (Opperman, 1995).

Currently, DNA sequences of segment D2-D3, Internal Transcribed Spacer-(ITS) of ribosomal RNA and Cytochrome oxidase subunit I-COI of mitochondrial DNA have been obtained for *B. cocophilus* in coconut palm and deposited in public molecular databases, which are used as a reference for the identification of the nematode (Ye et al., 2007; Silva et al., 2016). However, to date in Colombia no molecular characterization or phylogenetic analysis of *B. cocophilus* from oil palm has been performed.

In Tibu, oil palm plants with symptoms similar to those induced by the disease known as the red ring had been eliminated, to prevent their spread. However, palm growers have observed that some plants remain diseased for a longer time without showing the severity of the disease, so there is doubt about the diagnosis and identity of the causal agent, especially because sometimes the continuous ring is not observed or because the nematode is not found in sampling surveys. Due to the observations made by the palm growers, this research had as main objective the diagnostic of the disease by the verification of the symptoms, the identification of the nematode and confirmation of its identity at the morphometrical and molecular levels using diagnostic characters recommended and the amplification of the large subunit D2-D3 of the 28S.

## Materials and methods

### Symptoms observation and sampling

In total, 24 lots were visited located in 12 farms of Tibu (Norte de Santander), each of about 10 ha; in each plantation, plants with abnormal aspect were identified, and previously selected by the health censors (expert persons). In each case, description of the external and internal symptoms was recorded when the palms were knocked down with a chainsaw at the base of the stem and then cutting in sections to observe the internal symptoms. Similarly, cuts were made in petioles, bunches, buds, and spear leaf.

Symptomatic tissue samples as petioles, base of spear leaf, inflorescences, peduncle of inflorescences and bunches and stem from eradicated palms, were collected and kept in plastic bags, properly identified and refrigerated until processing. To collect tissues samples in palms that were not eradicated, a manual drill was used to perforate the stem at a height of approximately 1.5 m from the base at a depth close to 30 cm.

### Sample processing

Nematodes were extracted using the oxygenation-decantation method (Ravichandra, 2014). Five grams of tissue from each of the sampled organs were cutted into small portions and placed in a decantation sieve, with and without facial paper, rested on a decantation plate with enough water to cover the sample. After 24 h, the nematode suspension contained in the decantation plate was removed and concentrated at 20 mL with the 400-mesh sieve (Varón de Agudelo and Castillo, 2001).

### Quantification and nematode identification

To quantify the population of nematodes present in five grams of fresh tissue in each sample, three aliquots of 1 mL were taken and counting in chamber under a light microscope, Olympus PX40 and Olympus DP 73. For the morphological and morphometrical identification, the nematodes were killed with heat at 60°C for 4 min and fixated in 2% formalin. Then, semipermanent preparations were done and morphometric data were registered following Basil (1960), Brathwaite and Siddiqi (1975), Mai and Lyon (1975), Gerber et al. (1989). The morphometric data were taken using a compound microscope ZEISS Axio (China, microscope reference: 3136006171 Axio.A1).

### Molecular identification

DNA extraction was performed by the proteinase K method (Riascos-Ortiz et al., 2019). A specimen was divided into three parts with a sterile scalpel, transferring the sections to Eppendorf tubes with 15 µL lysis buffer (50 mM KCl, 10 mM Tris pH 8.0, 15 mM MgCl2, 0.5% Triton x – 100, 4.5% Tween – 20, 0.09% Proteinase K). Subsequently, the tubes were incubated at −80°C (15 min), 65°C (1 h), and 95°C (15 min), centrifuged at 16,000 g (1 min) and stored at –20°C. The PCR amplification of the expansion segment D2-D3 of the large subunit of ribosomal DNA (28S) was performed with the forward D2A primers: (5′-ACAAGTACCGTGAGGGAAAGTTG-3′) and reverse D3B: (5′-TCCTCGGAAGGAACCAGCTACTA-3′) according to De Ley et al. (1999). The PCR conditions were initial denaturation during 2 min at 94°C followed by 40 cycles of 45 sec at 94°C, 45 sec at 55°C and 1 min at 72°C and final extension of 10 min at 72°C. The PCR products were sequenced in both directions by the company Bionner (South Korea).

### Phylogenetic analysis

The sequences obtained were edited using the Geneious software (Kearse et al., 2012). Once the sequences edition were carry out, their identity was confirmed using the software BLASTn (http://www.ncbi.nlm.nih.gov/BLAST). Subsequently, the sequences presented under the accession numbers in Table 1 were aligned and analyzed using the MUSCLE algorithm included in the program MEGA6 (Tamura et al., 2013). Based on the matrix obtained, the nucleotide substitution model was determined by taking into account the Bayesian information criterion (BIC) using ModelGenerator v.0.851 software (Keane et al., 2006). The phylogenetic relationship was determinate by the maximum likelihood (ML) method based on the general time reversible (GTR) model, and the Gamma distribution, which was used to model the differences in evolutionary speed between locations. The internal support of the nodes was carry out using the bootstrap method with 1,000 replicates. *Aphelenchoides besseyi* sequence was used as external group (AY508109).

**Table 1.** Information of partial sequences D2-D3 of ribosomal DNA downloaded from GenBank and obtained in the present study for *Bursaphelenchus*.

Isolate	Species name	Location	Plant-host	Insect host	GenBank accession number	Reference or source
1	*B. cocophilus*	Tibu, Norte de Santander, Colombia	*Elaeis guineensis*	*R. palmarum*	MN612640	Present study
2	*B. cocophilus*	Tibu, Norte de Santander, Colombia	*Elaeis guineensis*	*R. palmarum*	MN612641	Present study
3	*B. cocophilus*	Tibu, Norte de Santander, Colombia	*Elaeis guineensis*	*R. palmarum*	MN612642	Present study
4	*B. cocophilus*	Tibu, Norte de Santander, Colombia	*Elaeis guineensis*	*R. palmarum*	MN612643	Present study
136	*B. abruptus*	USA	None	*Anthophora abrupta*	AY508073	Ye et al. (2007)
137	*B. abietinus*	Austria	*Pityokteines vorontzowi*	*Abies alba*	AY508074	Ye et al. (2007)
170	*B. anatolius*	Turkey	*Halictus* sp.	None	AY508093	Ye et al. (2007)
S12	*B. cocophilus*	Brazil	*Cocos nucifera*	*R. palmarum*	KT156772	Silva et al. (2016)
NT25	*B. cocophilus*	Colombia	*Cocos nucifera*	*R. palmarum*	KT156775	Silva et al. (2016)
NT26	*B. cocophilus*	Colombia	*Cocos nucifera*	*R. palmarum*	KT156776	Silva et al. (2016)
153	*B. fungivorus*	Germany	Greenhouse soil	Unknown	AY508082	Ye et al. (2007)
154	*B. hellenicus*	Greece	*Tomicus piniperda*	*Pinus brutia*	AY508083	Ye et al. (2007)
168	*B. mucronatus*	Germany	*Picea abies*	*Monochamus galloprovincialis*	AY508091	Ye et al. (2007)
173	*B. poligraphi*	Germany	*Picea abies*	*Polygraphus poligraphus*	AY508096	Ye et al. (2007)
171	*B. platzeri*	USA	None	*Carpohilus humeralis*	AY508094	Ye et al. (2007)
174	*B. seani*	USA	None	*Anthophora bomboides*	AY508097	Ye et al. (2007)
176	*B. seani*	USA	None	*Anthophora bomboides*	AY508099	Ye et al. (2007)
180	*B. sexdentati*	Italy	*Pinus pinaster*	Unknow	AY508103	Ye et al. (2007)
1057 J	*B. taphrorychi*	Poland	*Fagus sylvatica*	*Taphrorychus bicolor*	MF422699	Tomalak et al. (2017)
98	*Aphelenchoides besseyi*	USA	*Fragaria ananassa*	–	AY508109	Ye et al. (2007)

## Results

### Description of symptoms

During the sampling, 24 lots of 12 farms were visited, located in Punta de Palo, La Libertad, M14, M24, Oru L15, Playa Rica, Kilometer 15, Llano Grande, Refineria, and Campo Dos (Tibu, North Santander) for a total of 32 samples between stem, peduncle of bunches and inflorescences, petiolar base and spear leaf base. Palms with symptoms similar to those that have been associated with a red ring by many researchers were observed on all farms.

**Figure 1:**
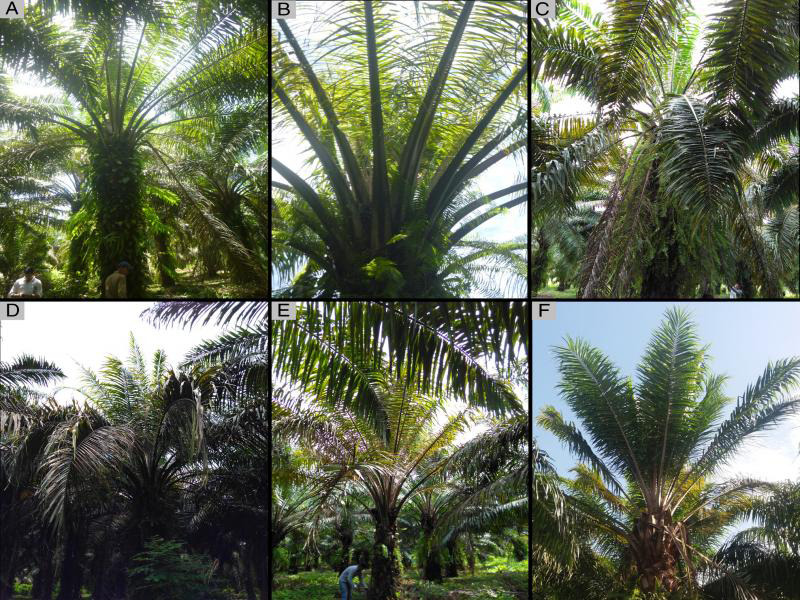
Foliar symptoms caused by *Bursaphelenchus cocophilus*, causal agent of red ring disease on oil palm. A: General aspect of diseased oil palm and some chlorosis on upper leaves. B: Thin leaflets and leaf chlorosis of the upper third. C: Shortening of young leaves. D-F: Progressive collapse of chlorotic leaves and leaf rotting.

**Figure 2:**
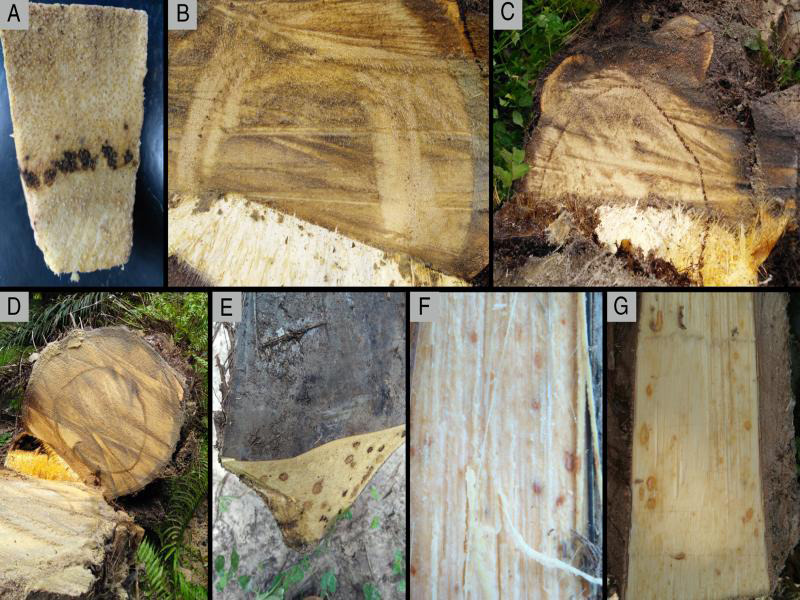
Symptoms caused by *Bursaphelenchus cocophilus*, the red ring nematode, in oil palm production in Colombia. A: Final piece of stem tissue from symptomatic and advance zone of the lesion. B: Small necrotic dots that form a discontinuous ring. C, D: Continuous necrotic area forming a ring of reddish brown color. E-G: Symptoms at the base of petioles of leaves.

The symptoms observed in diseased palms of Tibu were varied; the most frequent being chlorosis of the young leaves, thin leaflets, collapsed, and dry bottom leaves which remain adhered to the stem, accumulation of spear leaf, and short leaves. As the disease progresses the chlorotic leaves turn brown and foliar drying is observed (Fig. 1). In this study, some palms had advanced symptoms. In cross sections of the stem, the initial symptoms were characterized by small reddish-brown necrotic spots distributed in the vascular bundles (Fig. 2A, B). In more advanced stages of the disease was possible to observe the complete reddish brown ring of a few centimeters wide and near the periphery of the stem (Fig. 2C, D). The upper third leaves were shorter, chlorotic, and sometimes with thin, and dry leaflets. Necrotic points and sometimes large areas affected with necrotized tissue were observed when cross-sectional and longitudinal cutting of the petioles (Fig. 2E-G). When the sample was collected with the drill it was possible to observe in some palms small pieces of necrotic reddish tissue indicating that the palm had formed the ring.

### Quantification and identification of the nematode population

**Figure 3:**
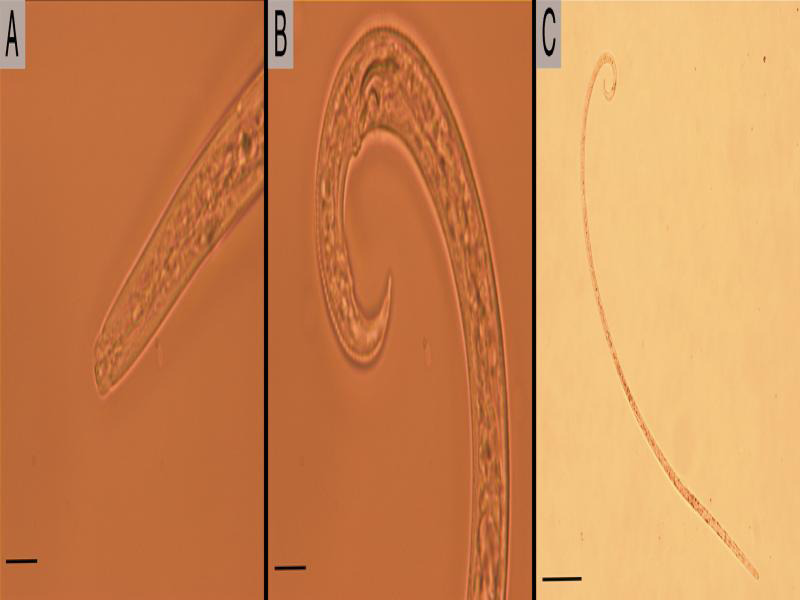
Microphotographs *Bursaphelenchus cocophilus*. A, B: Previous segment observed estomatostilet and male tail detail. Scale bar = 20 µm. C: Long slim male of *Bursaphelenchus cocophilus*. Scale bar = 100 µm.

The *Bursaphelenchus cocophilus* nematode (Fig. 3) was recovered from all tissues evaluated: stem, petiolar bases, inflorescences, bunch peduncle, and spear leaf base in variable populations (Table 2). A range of population from 720 to 1,295 individuals per 5 grams of stem tissue and petiolar base was found, respectively (Table 3). The nematode was recovered in almost all the sampled plants except in two palms, one of them had the external symptoms similar to those of the red ring; however, the ring was not observed internally and the nematode was not recovered in the stem tissue. On the other hand, the bud of this plant was decomposed and when cut it presented an aqueous substance of white color and foul odor. In the case of the second palm, the sampling was done with the drill and by this method it was not possible to confirm the presence of the ring; however, external symptoms were observed. Regarding the extraction method, it was possible to obtain the nematode with and without facial paper; however, the highest absolute frequency was obtained when the samples were processed without facial paper presenting values of 71.4% when the tissue came from eradicated palms and 85.7% of the tissue taken with a drill (Table 3).

**Table 2.** Average number of nematodes (*Bursaphelenchus cocophilus*) in five grams of fresh tissue from different plant organs according to extraction and sampling method.

	Extraction method (Decantation)		Sampling method	
Tissue class	With facial paper	Without facial paper	Palms knocked down	Palms standing (Drill)
Peduncle	10	8	X	
Petiolar Base	16	501	X	
Spear leaf base	5	0	X	
Inflorescences	110	–	X	
Stem	469	51	X	
Stem	1	4		X

**Table 3.** Population of *Bursaphelenchus cocophilus* according to sampling and extraction method.

	Extraction method (Decantation)		Sampling method	
	With facial paper	Without facial paper	Palms knocked down	Palms standing (Drill)
Maximum Population	1,295	720	X	
Maximum population	2	5		X
Absolute density	150	150	X	
Absolute density	0.86	4		X
Absolute frequency	58.8	71.4	X	
Absolute frequency	42.9	85.7		X

**Notes:** Absolute frequency = (No. of samples in which a genus was observed/total of evaluated samples) × 100. Absolute density = No. of average of individuals per five grams of fresh tissue.

From the samples processed in the present study, only were obtained juveniles and males from *B. cocophilus*. To morphological level the males were characterized to present appearance of thread with a thin stylet and sometimes with little visible basal knobs and a tail curved ventrally in a resting state. Because females were not observed, morphometric data were registered for the males. The values registered for the diagnostic characters recommended were similar to those reported in the original and reference descriptions of *B. cocophilus* (Table 4).

**Table 4.** Morphometric data of males of a *B. cocophilus* population characterized in the present study and others of reference populations

Locality/Publication	Tibu-North Santander, Colombia oil palm (Present study) *n*=12	Brazil Coconut Red Ring Disease After (Lordello and Zamith, 1954) *n*=5	Trinidad coconut Red Ring Disease (Basil, 1960) *n*=10	Venezuela oil palm Red Ring Disease (Gerber et al., 1989) *n*=10	Venezuela oil palm Little leaf (Gerber et al., 1989) *n*=10
Body length	964.67±70.07 (807.09-1062.67)	820-1420	1020 (840-1160)	1017±77 (841-1111)	866±71 (789-965)
*a*	83.89±10.68 (69.53-99.78)	92-143	120 (100-179)	129.7±13.3 (113.8-150.0)	99.3±11.8 (78.9-116.1)
*c*	29.33±1.72 (27.02-32.25)	22-47	28 (24-35)	26.1±2.2 (22.5-29.2)	22.4±2.8 (18.8-26.5)
*c′*	3.70±0.31 (3.19-4.31)	–	–	5.6±0.5 (4.8-6.4)	5.4±0.8 (4.7-6.8)
*T*	96.58±0.20 (96.30-96.90)	–	–	–	–
Max. Body diam.	11.62±1.34 (9.97-14.22)	–	–	8±1.0 (7.0-9.0)	9.0±1.0 (7.0-10.0)
Stylet	10.90±0.88 (9.07-12.12)	10.7-13.8	12-13	11±1.0 (11-12)	12.0±1.0 (11.0-12.0)
Lip region width	4.61±0.52 (3.81-5.22)	–	5.5	–	–
Lip region height	3.13±0.43 (2.43-365)	–	3.0	–	–
Median bulb length	12.16±0.79 (10.75-13.04)	–	–	–	–
Median bulb diam.	5.99±0.84 (4.66-7.64)	–	–	–	–
Tail length	32.83±2.35 (28.94-37.90)	–	–	39.0±4.0 (32.0-46.0)	39.0±4.0 (33.0-46.0)
Cloacal or anal body diam.	8.90±0.71 (7.75-10.16)	–	–	7.0±1.0 (5.0-8.0)	7.0±1.0 (6.0-9.0)
Spicule length	15.21±1.41 (13.29-17.32)	–	–	12.0±1.0 (11.0-13.0)	11±1.0 (10.0-13.0)

**Note:** Measurements in micrometers.

### Molecular characterization and phylogenetic analysis

**Figure 4:**
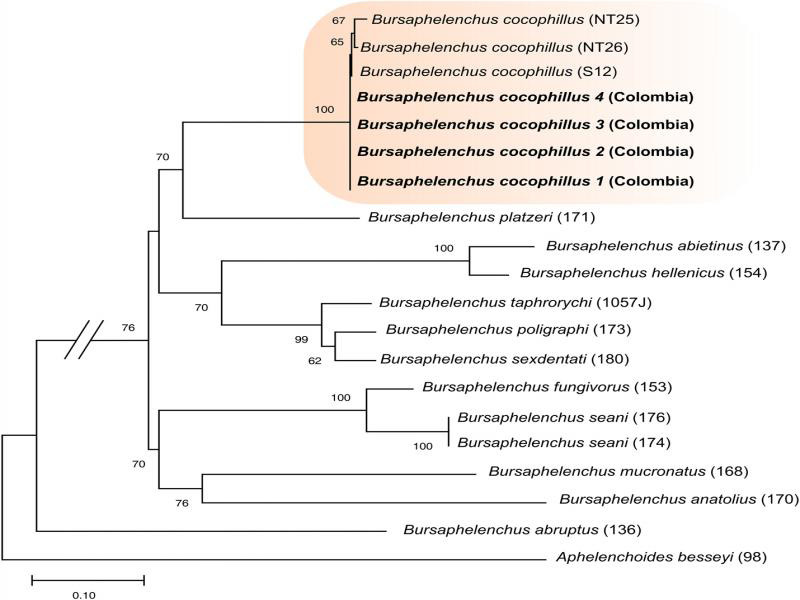
Phylogenetic tree obtained by the statistical method of maximum likelihood based on the general time reversible (GTR) model of the consensus sequences of the D2-D3 partial segment of some species of the genus Bursaphelechus. The sequences of this work are indicated in bold. The numbers on the nodes indicate bootstrap values >70%. The species Aphelenchoides besseyi (AY508109.1) is included as outgroup.

Four consensus sequences, with accession numbers MN612640 to MN612643, of segment D2-D3 were obtained, which presented a percentage of similarity between 99.5 and 100% with reference sequences of *B. cocophilus* previously deposited in GenBank (AY508076, KT156770, KT156772). The sequences of segment D2-D3 obtained in this study are the first of this amplicon reported for *B. cocophilus* in oil palm in Colombia. According to the phylogenetic tree of ML (Fig. 4), the *B. cocophilus* consensus sequences obtained in this study for D2-D3 grouped in the same clade with reference sequences of the same species recorded for coconut palm (KT156772 from Espírito Santo-Brazil, KT156775, KT156776 from Nariño-Colombia) with a 100% bootstrap support. Likewise, the clade of *B. cocophilus* is clearly separated from that of other species of the same genus.

## Discussion

The symptoms observed in diseased oil palms of Tibu, included the syndrome of short leaf (recognized in Central America in 1986 and associated with *B. cocophilus*), and red ring, which had correspondence with the descriptions done by different authors in Colombia as well as in other countries of Central America and the Caribbean (Thorne, 1961; Van Hoof and Seinhorst, 1962; Chinchilla and Richardson, 1987; Griffith, 1987; Chinchilla, 1992; Cuthbert, 1993; Calvache et al., 1995; Griffith et al., 2005; Giblin-Davis et al., 2010; Sullivan, 2013). The initial symptoms observed as small reddish-brown necrotic spots distributed in the vascular bundles, and the presence of the complete reddish brown ring with a few centimeters wide and near the periphery of the stem, as advanced symptoms coincides with those observed by Chinchilla (1992). These symptoms are characteristic of red ring in oil palm and coconut palm and some researchers indicate that the ring is due to the formation of tyloses or thickening of the cell that does not allow the passage of nutrients and water (Griffith, 1987). In coconut palm, the ring is confined to the walls of parenchymal cells in the stems, petioles and the bark of the roots (Griffith, 1987). Plants may have external symptoms of red ring but internally the ring may be absent, discontinuous or continuous, at the basal part or at the top of the stem (Chinchilla, 1992; Calvache et al., 1995; Giblin-Davis et al., 2010). The ring can vary in color from a red to pink from cream to dark brown and can reach three to five centimeters wide but may vary depending on the size of the plant (Griffith et al., 2005).

*Bursaphelenchus cocophilus* nematode was isolated from all tissues evaluated in different population densities in correspondence to registered data by Calvache et al. (1995). This author reported that in palms with short leaves the nematodes are found in the spades of the floral primordia and at the base of the spear leaf. In palms with initial symptoms, the nematodes is not found in the stem or in the meristem; in palms with advanced or intermediate symptoms it is found at the base of the spear leaf and leaves, and stem, but not in the petioles, flower peduncles, roots, and soil.

The nematode was recovered in almost all the sampled plants except in two palms. Several authors indicate that external symptoms are not enough to diagnose the disease, due to it may be caused by other pathogens or factors such as nutritional deficiencies or mechanical damage, so it is necessary to obtain samples and verify the presence of the nematode (Dean, 1979; Chinchilla, 1991). It is important to consider that when the palm is knocked down it is possible to observe internal symptoms in detail and take tissue samples at the different sites of the plant. Through of the drill method was not possible to confirm the presence of the ring in plants with external symptoms and the nematode only was recovered in samples taken in the stem in a low density, possibly because a very low population of the nematode or because the tissue was in an advanced state of necrosis. The drilling method is faster, but the ring is not always observed, therefore, the use of this technique may lead to underestimating the population level or absolute density of the nematode. In coconut palm, the punched borehole methodology was used by the Colombian Agricultural Institute (ICA), for the diagnostic and eradication of palms with a red ring during the 70s and 80s years (Victoria et al., 1970).

According with the extraction method, the nematode presented the highest absolute frequency when the samples were processed without and with facial paper with values of 71.4 and 85.7%, respectively. The advantage of facial paper is that it allows obtaining a cleaner sample with few tissue residues, which facilitates the microscope nematode observation.

The maximum population of nematode was found in the stem and the morphological and morphometrical characteristics match with those recorded for the species *B. cocophilus* (Lordello and Zamith, 1954; Basil, 1960; Brathwaite and Siddiqi, 1975; Mai and Lyon, 1975; Gerber et al., 1989). The sequences of D2-D3 expansion segment had a similarity between 99.5 and 100% with reference sequences of *B. cocophilus* (AY508076, KT156770, KT156772). Considering, the evolutive relationships through the phylogenetic tree, the sequences of the nematode grouped in the same clade with others reference sequences of *B. cocophilus*. These results confirm that *B. cocophilus* is associated with symptomatic oil palms for red ring disease in the study area (Ye et al., 2007; Silva et al., 2016).

In conclusion, the red ring disease of the oil palm in Tibu (North Santander) presents the same external and internal symptoms described in Colombia and other countries where the disease has been registered, even when they were very varied. It was possible to extract and identify the *Bursaphelenchus cocophilus* nematode in the samples collected in Tibu, which confirms the diagnostic of the disease in this area. *B. cocophilus* was found in almost all tissues evaluated indicating that the nematode may be present in most of the plant’s organs. The sampling methods allowed the parasite to be recovered; however, sampling in eradicated palms allows a better diagnostic. Morphometric diagnosis integrated with molecular and phylogenetic analysis of the D2-D3 segment of ribosomal DNA confirmed that *B. cocophilus* is associated with red ring disease in oil palm crops in the study area.
